# A Review on the Use of Deep Eutectic Solvents in Protection Reactions

**DOI:** 10.3390/molecules29040818

**Published:** 2024-02-10

**Authors:** Rosa Scarpelli, Renata Bence, Natividad Carolina Herrera Cano, Antonio Procopio, Daniel Wunderlin, Monica Nardi

**Affiliations:** 1 Dipartimento di Scienze della Salute, Università Magna Græcia, Viale Europa, Germaneto, 88100 Catanzaro, Italy; procopio@unicz.it (A.P.); monica.nardi@unicz.it (M.N.); 2 ICYTAC, CONICET and Departamento Química Orgánica, Facultad de Ciencias Químicas, Universidad Nacional de Córdoba, Ciudad Universitaria, Bv. Juan Filloy s/n, Córdoba 5000, Argentina; rbence@unc.edu.ar (R.B.); daniel.wunderlin@unc.edu.ar (D.W.)

**Keywords:** deep eutectic solvents, green chemistry, protection reactions, esters, amides

## Abstract

Given the recent research on the application of eco-sustainable methods in organic chemistry, we have focused our attention on the derivatization processes for fundamental functional groups in organic chemistry, such as amino, hydroxyl and carbonyl groups. Protection reactions are needed to temporarily block a certain reactive site on a molecule. The use of green solvents in this context has made an excellent contribution to the development of eco-sustainable methods. In recent years, deep eutectic solvents (DESs) have had great success as a new class of green solvents used in various chemical applications, such as extraction or synthetic processes. These solvents are biodegradable and nontoxic. In this framework, a list of relevant works found in the literature is described, considering DESs to be a good alternative to classic toxic solvents in the protection reactions of important functional groups.

## 1. Introduction

In organic chemistry, synthetic reactions require the use of reagents and catalysts to obtain the formation of new products, sometimes also requiring the use of appropriate protecting groups to prevent the formation of undesirable bonds and secondary reactions [1]. In the planning of a synthetic process, the protection of functional groups within a polyfunctional molecule plays a key role, and the choice of suitable protecting groups can affect the efficiency and success of the synthesis [2]. Functional groups are particularly susceptible to a wide variety of reagents, both oxidizing reagents and alkylating reagents. The introduction and successive removal of a protecting group during a synthetic sequence requires the use of organic reagents and solvents. The use of toxic solvents and the formation of large quantities of industrial waste constitute serious problems for the environment and human health [3,4].

Environmental pollution poses a massive threat to the planet. To tackle these issues, a new strategy, called the European Green Deal (EGD), was presented by the European Parliament in November 2019. The EGD includes a series of initiatives, strategies and legislative acts that aim to move the EU onto the path of a green transition, reduce the emission of greenhouse gases and support energy from clean sources [5]. In this respect, Green Chemistry has acquired a crucial role in the industrial sector, proposing a new philosophy of “clean work” that aims at the development of an ecologically conscious community, in which the sustainability of raw materials is the basis of industrial synthetic processes. Among the 12 principles of Green Chemistry, set out by Anastas, is the use of solvents that are more environmentally friendly [6,7].

Chemical solvents are widely used in the chemical, pharmaceutical, petrochemical and textile industries. Considering the often-dangerous nature of these products, the use of traditional organic solvents requires extremely careful handling. Not only are they not eco-friendly but they also have a high level of toxicity and volatility, and their use poses very serious and irreparable environmental risks [8,9].

The use of environmentally friendly solvents in various synthetic processes certainly contributes to a reduction in the formation of environmentally hazardous industrial waste, as well as ensuring greater safety at work. Solvents designated as “green solvents”, such as subcritical water, supercritical fluids, ionic liquids, DESs, NaDESs and HDESs, are all characterized by excellent properties, posing little to no toxicity to human health, to animal health, to plant species [10] or to the environment, being more sustainable and bio-renewable than the already existing hazardous solvents. Deep eutectic solvents (DESs) represent a valid alternative to conventional organic solvents due to their simple preparation using natural and easily accessible compounds [11]. They are most promising and excel even over biomass-derived solvents, and their versatility and ease of preparation have caused them to gain a lot of attention, especially in the chemical synthesis field.

DESs are defined as systems composed of a binary mixture of two components—a hydrogen bond acceptor (HBA), including choline chloride and betaine as quaternary ammonium, sulfonium or phosphonium salts and metal halides, and a hydrogen bond donor (HBD), including urea, renewable carboxylic acids, polyols and saccharides—which are capable of self-associating to form a new eutectic phase with a melting point lower than the individual melting points of their constituent components. The production of DESs from natural sources, together with almost no toxicity, total biodegradability and a low cost, makes these solvents suitable for extraction and chemical synthesis processes [12,13,14,15].

Depending on the type of DES complexing agent, there are four/five types of DESs: (I) quaternary ammonium salts with an anhydrous metal chloride; (II) quaternary salts with a metal chloride hydrate; (III) quaternary ammonium, sulfonium or phosphonium salts (HBA) with an HBD compound; (IV) metal halides with an HBD; (V) “non-ionic DESs”, which are those in which both components are molecular substances. “Natural” DESs or NaDESs are instead considered a subclass of type IV DESs. The term was coined to distinguish liquids made up of primary or secondary metabolites of cells; this means that NaDESs are solvents prepared using natural components from cell metabolism.

In consideration of the role of protecting groups in synthetic processes and the growing problem of environmental pollution, this review proposes a compilation of all the strategies implemented over the years that provide for the use of environmentally friendly solvents in functional group protection reactions.

In the following paragraphs, the protection methods of the two most important functional groups in organic synthesis will be reported, the amino and hydroxyl groups, which involve the use of DESs.

## 2. Green Solvents in Green Chemistry

### 2.1. Protection Reactions Using Deep Eutectic Solvents

Two of the most important steps in synthetic organic chemistry routes are the protection and deprotection of important functional groups. In multifunctional compounds, when a chemical reaction is to be carried out selectively at one reactive site, other reactive site must be temporally blocked. Protecting groups are used in synthesis to temporarily mask the characteristic chemistry of a functional group because it interferes with another reaction. A good protecting group should be easy to implement and easy to remove in high-yield reactions and inert to the conditions of the reaction required.

The pharmaceutical industry has always been very attentive to this field of organic sites because the synthesis of many drugs using multifunctional molecules requires the use of protecting groups. In this regard, the ACS Green Chemistry Institute^®^ Pharmaceutical Roundtable (GCIPR) (Washington, DC, USA) has produced a Reagent Guide to inform and guide chemists toward the use of greener reagents for various chemical transformations.

Green solvents have shown great promise as alternatives to or replacements for the classic volatile organic solvents. In this review, the derivation methods of the most important functional groups using deep eutectic solvents as the reaction media will be reported.

#### 2.1.1. Amine Protection Using Deep Eutectic Solvents

Amine group protection is an important topic in organic chemistry because protected amines have numerous applications as drug molecules, materials, agricultural chemicals and natural products [16].

Amines are nucleophilic and basic in nature and are particularly susceptible to various chemical reagents: in the presence of an acid, salts are formed; in the presence of carboxylic acids, amides are produced; in the presence of an alkyl halogenide, an alkylamine is formed. Therefore, various methods have been developed to protect the amine group in order to make a chemical reaction more selective. The most frequently used amino-protective groups are 9-fluorenylmethoxycarbonyl (Fmoc) and tert-butyloxycarbonyl (Boc), both of which inhibit the reactivity of the amine group by transforming it into a urethane derivative and an amide, respectively. However, complex procedures requiring the use of toxic solvents and the generation of reaction residues are reported in the literature for the protection of amine groups [16,17].

The necessity of adopting more environmentally friendly and sustainable technologies has led to the development of new synthetic methods in organic chemistry [18].

Among the first reactions involving the amino groups using green solvents, the synthesis of cyclopentenones starting from morpholine achieved considerable interest [19,20,21,22]. An effective and rapid method for the synthesis of bifunctionalized cyclopentenones in an eco-friendly and biodegradable DES based on a ChCl–urea mixture has been developed [23]. The recyclability of the DES in the reaction of furfural with morpholine is evaluated. The reaction mixture, including ChCl–urea and its products, was extracted using ethyl acetate (an extractive procedure repeated two more times). The next reaction cycle was performed by simply adding new fresh reagents to the same DES. The method has a good level of generalizability and is applicable to various 4,5-bisubstituted as well as 2,4-bisubstituted cyclopentenones. Of note, in the present method, no catalyst or base was used, and the use of any toxic organic solvent was avoided.

The same green solvent is used as the catalyst and reaction media in an eco-sustainable method for the synthesis of symmetric organic carbonates and ureas [24]. The applicability of this green synthesis method was also explored for the reaction of nucleophiles such as phenyl hydrazine and phenyl glycine with dimethyl carbonate, obtaining good yields of the products.

The procedures for the synthesis of benzimidazoles have become a focus in synthetic organic chemistry, as they are building blocks of great interest for the development of compounds with pharmacological activity (i.e., antimicrobial, antiviral, anti-inflammatory, analgesic, etc.), and some of the already synthesized compounds have found very strong application in medicine praxis. In this context, the use of deep eutectic solvents (DESs) both as reaction media and reagents without any external solvents provides markable advantages in terms of the yields as well as in the work-up procedures of the reactions [24].

Di Gioia et al. decided to explore the potential solvent/reagent double role of DESs as an efficient way to minimize waste formation. The use of urea to form type III eutectic solvents where choline chloride is mostly taken as the quaternary ammonium cation has been known for a considerable time but can be applied to a wide variety of other hydrogen bond donors (HBDs), such as organic acids, alcohols and amines. In light of the above statement, *o*-phenylendiamine was explored as an HBD to combine with choline chloride (at a1:1 molar ratio), thus forming a eutectic mixture in which the diamine would therefore be part of the solvent and reactant at the same time. A differential scanning calorimetry (DSC) analysis of the mixture was performed, as well as for the individual components, to demonstrate the formation of the DES. ChCl and *o*-phenylendiamine are solid components melting at 302 °C and 102 °C, respectively: the DSC analysis of the mixture resulted in a eutectic that melts at 32 °C. This result demonstrated the successful formation of a eutectic with a melting point significantly lower than either of the individual components. The new reactive DES obtained is applicable to the chemical synthesis of monosubstituted benzimidazole derivatives (using 1 mmol of aldehyde corresponding vs. HBD) and in the same reaction conditions but using 2 mmol of aldehyde vs. HBD to the synthesis of 1,2-disubstituted benzimidazoles [25].

After the completion of the reactions, ChCl is dissolved in water and can be recycled easily using water distillation under a vacuum, demonstrating the potential industrial applicability of this green procedure.

**Figure 1:**
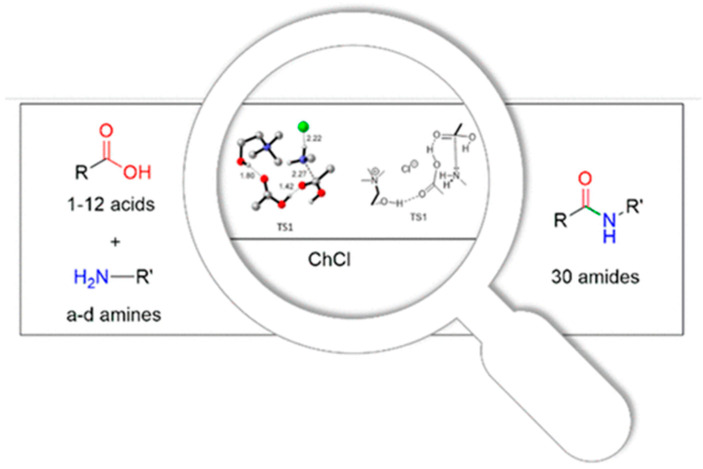
Choline chloride’s role in synthesis: the case of direct amidation [26].

At the same time, the use of ChCl as a promoter for the direct amidation of carboxylic acids has been developed (Figure 1). The procedure is performed using microwave (MW) irradiation. In this regard, it has been verified that the reaction benefits from the specific MW effect, showing a thermal effect in which the nature of the reagents and the mechanism of the reaction allows the effective accumulation of the MWs. Various microwave (MW)-assisted amidation tests were carried out using primary, secondary, benzyl and aromatic amines. ChCl’s role in the stabilization of the reaction intermediates and the coordination of the two reagents was reported as investigated using FT-IR analysis and DFT calculations. The amide (with a98% yield) is obtained in 18 min [26].

The use of ChCl in an amidation reaction and its efficient role were tested recently in the selective protection of amines and the cleavage of the fluorenyl methoxycarbonyl group (Fmoc) [27]. In particular, one study shows DESs can be used in classic reactions in the protection of amines. In the first phase of the study, preliminary experiments were performed using aniline (1 mmol) and Fmoc chloride (1 mmol) in a DES system based on choline chloride. Different combinations of eutectic choline chloride mixtures were used (ChCl/urea; ChCl/glycerol; ChCl/lactic acid). Using the DES based on choline chloride/urea, low results were obtained when the reaction was conducted at room temperature (10% in yield), with a more efficient yield (quantitative) at a temperature of 80 °C after only 5 min. Instead, very good results (90% yield) were obtained with the DES based on choline chloride/lactic acid after 10 min at 80°.

**Figure 2:**
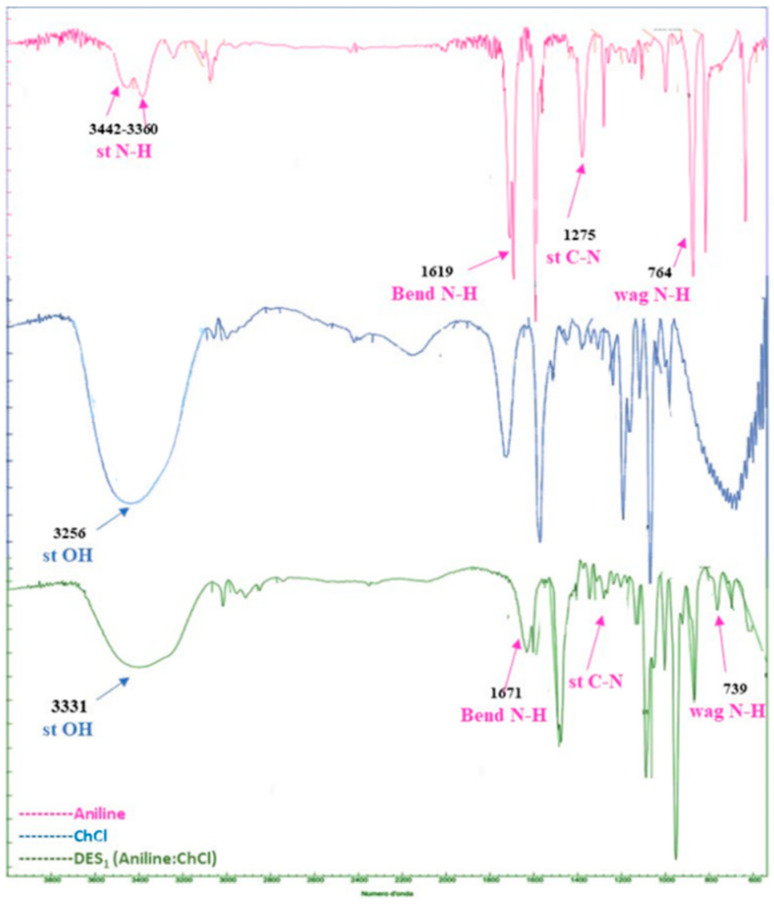
FT-IR spectra of aniline (pink), choline chloride (blue) and aniline: choline chloride mixtures (green) [27].

In order to further improve the green process, the next approach was to form a eutectic mixture based on aniline (HBD) and ChCl (HBA). As demonstrated using Fourier transform infrared spectroscopy (FT-IR) (Figure 2) and nuclear magnetic resonance (NMR) analyses, choline chloride combines with the different amines to form deep eutectic mixtures.

In the first phase, a deep eutectic solvent of ChCl: aniline (at a1:1 molar ratio) was prepared by heating the two components at 80 °C for 2 h to obtain a light yellow liquid product.

Subsequently adding Fmoc-Cl to this eutectic mixture and heating it to 60°, after a few minutes, obtains a good yield (quantitative) of the protected amine, and after the simple addition of water, precipitation of the crude product occurs.

**Scheme 1:**
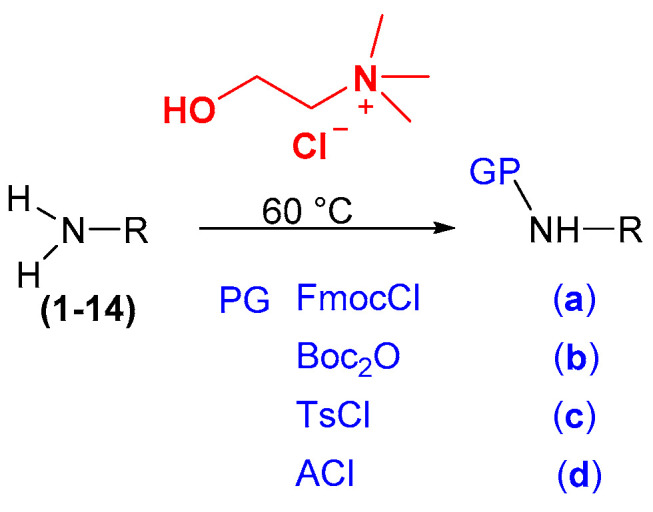
*N*–protection in amine-based DESs [27].

Once the protocol had been optimized, the second phase of the study was to react different anilines and substituted amines with different protecting groups [Fmoc (9-fluorenylmethoxycarbonyl), Boc (*t*-butyloxycarbonyl), Ts (*p*-toluenesulphonyl) and Ac (acetyl)] (Scheme 1).

It is observed how aniline and its derivatives were easily protected, leading to the formation of protected amines with excellent yields.

**Figure 3:**
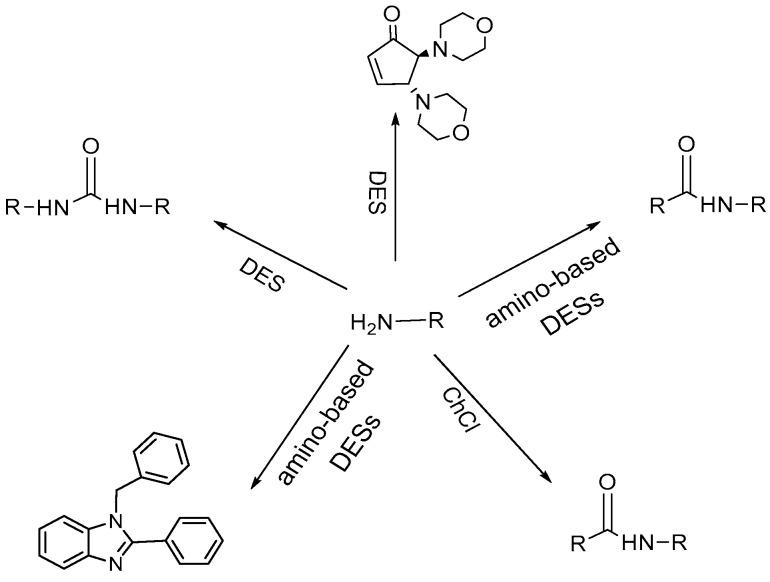
Derivatization of amine groups using DESs as green solvents.

The research demonstrated how it is possible to conduct amine group protection reactions using an environmentally friendly method that allows the formation of amide bonds in situ with excellent yields (Figure 3).

#### 2.1.2. Derivatization of Alcohol Using DESs

The protection of hydroxyl groups is of fundamental importance in almost every multi-step synthetic approach to complexing molecular architectures of industrial interest, such as (glyco)peptides [28], oligosaccharides [1,29], nucleotides [30,31,32,33] and/or active pharmaceutical ingredients (APIs) [34,35]. In fact, the incorporation of an OH-protecting group is frequently indispensable to prevent unwanted reaction pathways being triggered by the acidic and nucleophilic nature of the hydroxyl functionality [36].

**Scheme 2:**
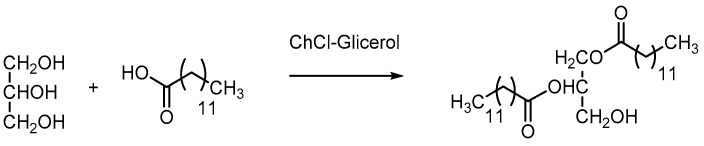
Esterification reaction of glycerol with lauric acid using ChCl–glycerol.

In a study conducted by Abbot et al. in 2010, the chemical properties of ChCl–glycerol mixtures were explored for the esterification of glycerol with lauric acid (Scheme 2) [37]. The findings revealed that the addition of ChCl promoted the reaction toward the diester product, in contrast to the pure glycerol system, which favored monoester formation.

It was proposed that ChCl disrupts the three-dimensional structure of the glycerol, thereby promoting esterification reactions with the pre-existing glycerol molecules. Furthermore, ChCl may interact with the water molecules generated during the reaction, displacing them from equilibrium and accelerating the overall process.

**Scheme 3:**
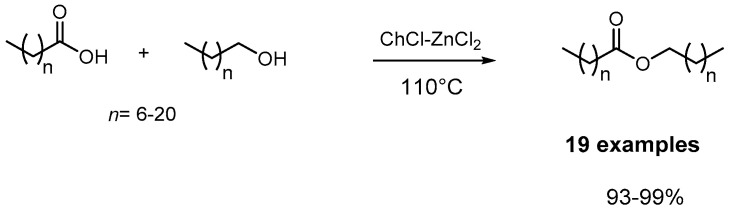
Esterification reaction of glycerol with lauric acid with [ChCl][ZnCl_2_]_2_.

**Scheme 4:**
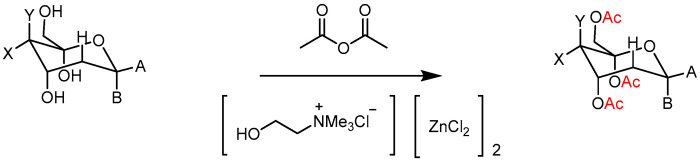
O-acetylation of monosaccharides with [ChCl][ZnCl_2_]_2_.

While Lewis- or Brønsted-acid-catalyzed esterification is a well-established protocol in organic synthesis, challenges arise when dealing with acids or alcohols possessing car-bon chains longer than C10. In addressing these challenges, a DES formed of choline chloride (ChCl) and zinc chloride (ZnCl_2_) at a 1:2 molar ratio emerged as an effective catalytic medium for the esterification of aliphatic carboxylic acids and long-chain alcohols (C8-C22) (Scheme 3) [28]. Notably, this DES exhibited the added advantage of recyclability, demonstrated through six successive reuses in the esterification of octanoic acid and cetyl alcohol without a reduction in activity. The same DES was successfully employed in the acetylation of monosaccharides (Scheme 4) [38].

The protocol was applied to the preparation of per-*O*-acetylated hemiacetals of aldoses that, on further heating to 100 °C, underwent selective deacetylation at the anomeric position [39]. The operational simplicity, no further purification steps and the recyclability of the DES are key features of this protocol.

In contrast, a DES formed of trimethylcyclohexylammonium methanesulfonate and *p*-toluenesulfonic acid (TCy-AMsO/pTsOH at a molar ratio of 1:1) was identified as a suitable catalytic reaction medium for smaller alcohols (less than C8) [40].

**Scheme 5:**
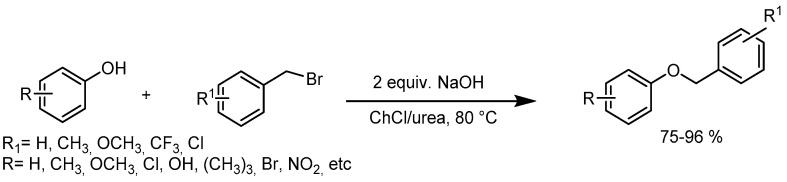
Benzylation of phenols with benzyl bromide using a deep eutectic solvent (choline chloride/urea).

Singh and coworkers [41] explored the application of this DES to the benzylation of phenols with benzyl bromide, revealing the promising solvent and phase transfer medium capabilities of ChCl/urea (Scheme 5). Lower yields were obtained in other media, such as ChCl/glycerol and ChCl/ZnCl_2_. The study demonstrated the versatility of this methodology, achieving the benzylation of structurally diverse phenols with different functional groups and various aromatic bromides and with good to excellent yields.

Shifting focus to the formation of silyl ethers from hydroxyl groups, this process finds wide-ranging applications, including the production of volatile derivatives suitable for gas chromatography (GC) and gas chromatography–mass spectrometry (GC–MS). Additionally, silylation acts as a protective measure in the synthesis of pharmaceuticals, steroids, sugars and natural products, while also facilitating surface modification for materials like clay and wooden objects [42,43,44].

Due to their ease of formation, resistance to oxidation, good stability toward most non-acidic reagents and easy deprotection in providing free alcohols, silyl ethers are popular and extensively used as the protecting groups for alcohols [45].

**Scheme 6:**
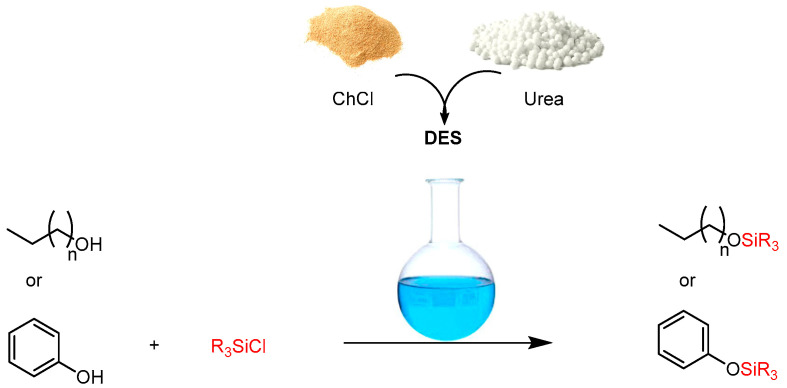
Base- and catalyst-free preparation of silyl ethers in DES.

A highly efficient and environmentally friendly procedure for silyl ether preparation was developed using a DES composed of choline chloride and urea (ChCl/urea) as the benign reaction medium. Silylation was achieved using trimethylsilyl chloride (TMSCl), triethylsilyl chloride (TESCl) and *t*-butyldimethylsilyl chloride (TDSCl) as the silylating agents (Scheme 6). This green protocol offers advantages such as high yields, operational simplicity, short reaction times and the elimination of environmentally hazardous solvents, bases and catalysts. Furthermore, its applicability to large-scale industrial silylation applications enhances its practical significance [46].

**Figure 4:**
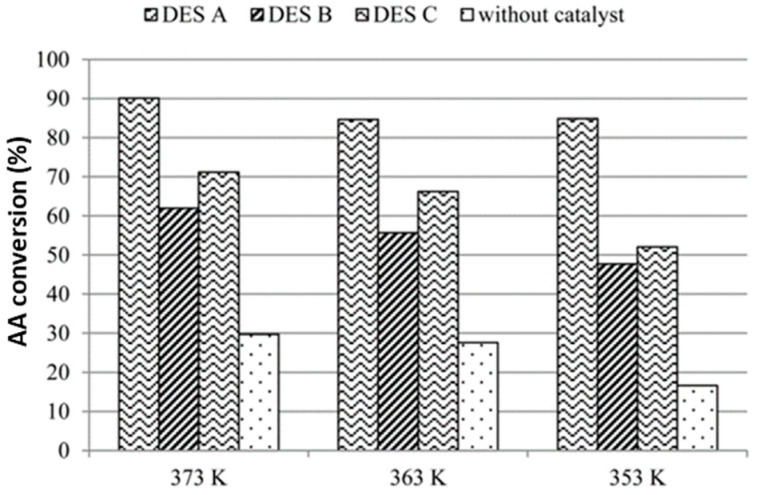
Comparison of the catalytic activities of the synthesized DESs in the esterification of acetic acid (AA) with 2-ethyl hexanol.

Taysun et al. delved into the use of Brønsted-type DESs in the esterification of acetic acid with 2-ethyl hexanol. The catalytic activity of benzyl triethylammonium chloride (BTEAC)-based DESs was investigated, with BTEAC serving as the hydrogen bond acceptor and pTSA, citric acid (CA) and oxalic acid (OA) as hydrogen bond donors. Various DESs were prepared, and for a 10 wt% of the DES, an alcohol:acid ratio 1:1 and a reaction time of 180 min, DESA (BTEAC-pTSA) provided the highest conversions at the different temperatures tested (Figure 4). The acidity of DESA was identified as a key factor contributing to its high conversion values and shorter reaction times, presenting a notable advantage over solid catalysts [47].

In recent decades, biocatalysis has made significant strides and become indispensable in the production of pharmaceuticals, industrial chemicals and biofuels [48]. However, its application is often hindered by stringent reaction conditions and the limited solubility of substrates in aqueous solutions. An effective approach to dealing with insoluble substrates involves utilizing enzymes.

**Scheme 7:**
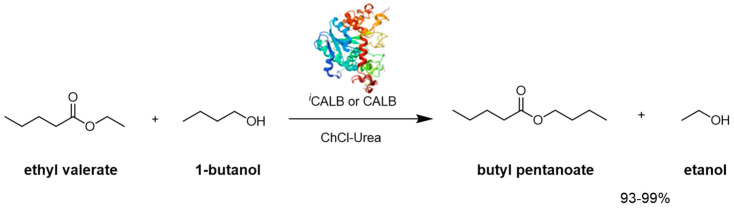
Transesterification reaction of ethyl valerate with 1-butanol using CALB and the immobilized form (iCALB).

The utilization of hydrophobic organic solvents, supercritical fluids, ionic liquids (ILs) and solvent-free media in biocatalytic reactions has provided compelling evidence that enzymes can maintain their activity and stability in environments traditionally deemed “unnatural” [49]. Pioneering investigations by Gorke et al. delved into biotransformation within deep eutectic solvents (DESs), either as solvents or cosolvents, specifically focusing on hydrolase-catalyzed transesterification. The model reaction centered around the transesterification of ethyl valerate with 1-butanol (Scheme 7) [50].

Both CALB and its immobilized form, iCALB, exhibited catalytic activity across all eight DESs tested, demonstrating conversions comparable to those observed in toluene for five of the DESs. Gorke et al. were instrumental in showcasing that urea, functioning as a robust hydrogen bond donor (HBD), when paired with choline chloride (Ch) as a hydrogen bond acceptor (HBA), did not induce the denaturation of the hydrolases, leading to esterification product yields ranging from 93% to 99%, both using CALB and iCALB.

The research conducted by Sua et al. unveiled that CALB effectively catalyzes transesterification reactions in pure DESs, with the efficiency markedly influenced by the composition and properties of the DESs employed. Hydrophobic DESs, particularly those based on terpenes, exhibited a more favorable impact on CALB’s activity compared to hydrophilic DESs.

Moreover, the study demonstrated that the catalytic efficiency of CALB in hydrophilic DESs could be augmented by increasing the quantity of hydroxyl groups in the DES components. In contrast, the presence of carboxyl groups and quaternary ammonium salts in the hydrophilic DESs hindered or deactivated CALB. Interestingly, the presence of carboxyl groups in the hydrophobic DESs exhibited a beneficial effect, preserving CALB’s natural structure and activity. Additionally, the presence of longer carbon chains in the DES components exhibited a positive correlation with CALB’s catalytic activity.

The research also identified a negative correlation between the hydrogen bond basicity of the DES and CALB’s transesterification activity, suggesting that systematic DES design could enhance CALB’s performance.

**Scheme 8:**
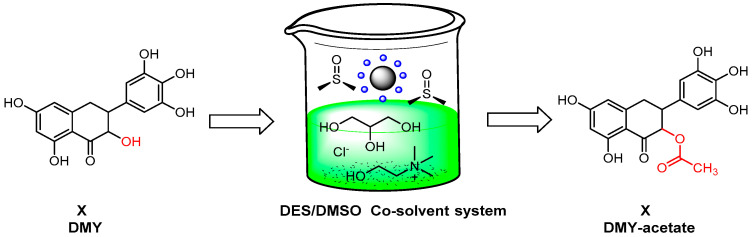
Enzymatic acylation of dihydromyricetin (DMY) using an immobilized lipase with a DES and DMSO cosolvent system.

Wen-Yong Lou’s research demonstrated the superiority of a cosolvent mixture containing choline chloride, glycerol-based DESs and dimethyl sulfoxide (DMSO) for enzymatically acylating dihydromyricetin (DMY) compared to traditional organic solvents [51] (Scheme 8). This innovative cosolvent system proved effective in DMY acylation and enhanced the synthesis of ester derivatives of polar polyhydroxylated compounds. DMSO played a pivotal role in improving the enzymatic acylation by enhancing the interaction between the enzyme and substrates, while choline chloride and glycerol-based DESs aided in dissolving the polar substrates and preventing enzyme inactivation in highly polar organic solutions. The cosolvent outperformed traditional organic solvents, offering significant advantages in terms of the enzyme stability and purification procedures.

Moreover, a novel cosolvent system, combining a DES and DMSO, was successfully employed for the first time as the reaction medium for the enzymatic acylation of DMY, catalyzed using immobilized lipase from *Aspergillus niger* (ANL). The optimal cosolvent, consisting of choline chloride and glycerol at a 1:3 ratio with DMSO (*v/v*), demonstrated impressive results, with an initial reaction rate of 11.1 mmol/h and a DMY conversion rate of 91.6%. The immobilized lipase, ANL@PD-MNPs, remained stable and reusable, maintaining a 90% conversion rate even after five usage cycles. Additionally, the resulting DMY-16-acetate exhibited a significantly higher lipid solubility compared to the raw DMY material, showcasing excellent antioxidative activity. This research underscores the potential of the DES–DMSO cosolvent as a promising alternative for DMY ester synthesis in scientific applications.

Blangetti et al. presented a study showing that acidic natural deep eutectic solvents (NADESs) are effective in catalyzing the tetrahydropyranylation of alcohols under mild conditions. This method enables the synthesis of tetrahydropyranyl (THP) ethers from various types of alcohols quickly, with easy isolation using the eco-friendly solvent cyclopentyl methyl ether (CPME) [52]. Remarkably, the reaction maintains excellent selectivity in the presence of competing hydroxy groups and can accommodate acid-sensitive functional groups.

**Scheme 9:**
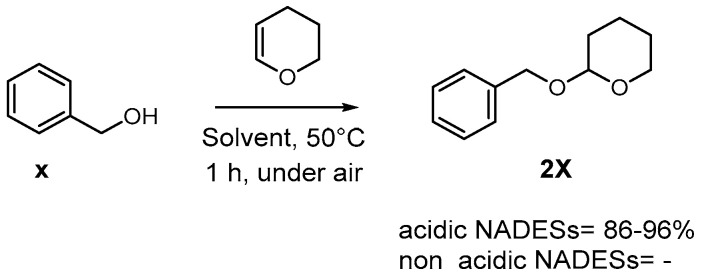
Tetrahydropyranylation of benzyl alcohol.

Their initial experiments used phenylmethanol as a model substrate, and the results confirmed the remarkable catalytic activity of the acidic NADESs. Adjusting the temperature or reagent quantities influenced the reaction yield. While other NADESs with different acid components produced slightly lower yields, they were still reasonable (86–96%) (Scheme 9).

Non-acidic NADESs based on glycerol, water or urea proved ineffective for this reaction. Additional investigations revealed the necessity of an acidic component in the NADES’s composition to promote the tetrahydropyranylation reaction.

In summary, this research highlights the effectiveness of acidic NADESs as green and efficient media for the tetrahydropyranylation of alcohols, with potential applications in the synthesis of THP ethers.

**Scheme 10:**
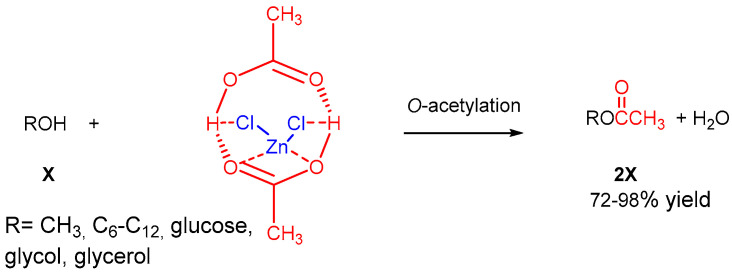
ZnCl_2_:2HOAc as a liquid complex and DE-acetylating agent of alcohols.

Fatemeh Tamaddon and Rashidi [53] demonstrated the effectiveness of ZnCl_2_:HOAc (molar ratio of 1:2, 0.2 mL) at 60 °C as a versatile acetylating agent for a wide range of alcohols and diols and glucose under optimized conditions (Scheme 10). Notably, ZnCl_2_:2HOAc demonstrated high chemo-selectivity in acetylating secondary alcohols over tertiary ones. Additionally, it proved suitable for the selective *O*-acetylation of diols and triols and per-*O*-acetylation of glucose, resulting in the desired mono-acetylated products. Even in cases involving aniline and Bu-NH_2_, ZnCl_2_:2HOAc only engaged in an acid base reaction with these compounds, highlighting its chemo-selectivity.

To further validate its chemo-selectivity, a competition acetylation reaction between Bu-NH_2_ and Bu-OH showed 100% selectivity for butyl acetate formation. The purity of the produced esters and butyl-ammonium acetate was confirmed using FT-IR spectra.

ZnCl_2_:2HOAc is presented as a liquid complex and the DE-acetylating agent with ZnCl_2_ and HOAc as the hydrogen bond acceptor (HBA) and hydrogen bond donor (HBD) components, respectively. The formation of this deep eutectic solvent (DES) was evidenced by changes in the melting point, density, viscosity and FT-IR vibrational modes due to the strong molecular interactions between the HOAc HBDs and chloride HBAs. This agent offers an efficient, cost-effective and eco-friendly alternative for acetylation reactions, particularly for the large-scale Friedel–Crafts acetylation of active phenols, long-chain acetate ester synthesis and the selective acetylation of hydroxyl groups over amino groups. It comes with the added advantages of high activity, atom efficiency, non-corrosiveness and reduced toxicity compared to conventional acetylating agents.

#### 2.1.3. Synthesis of Acetals Using DESs

**Scheme 11:**
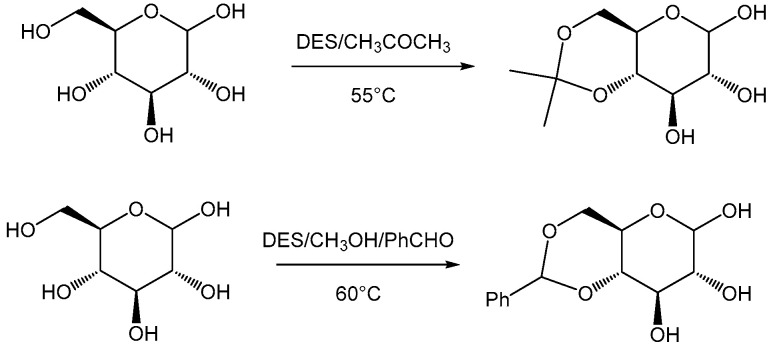
O-isopropylidenation, glycosidation and O-benzylidenation of monosaccharides.

The selective protection of the hydroxyl groups of monosaccharides is a very important reaction in organic chemistry. Particularly, O-isopropylidenation, glycosidation and O-benzylidenation are fundamental reactions widely used in synthetic carbohydrate chemistry [54,55]. Rokade et al. reported a one-pot synthesis of per-*O*-acetylated hemiacetals from free sugars [49] and the Ferrier reaction of glycals in a DES [56]. The same authors described herein a simple and convenient method for the synthesis of O-isopropylidene sugar derivatives and methyl O-benzylidene glycosides from free sugars using a DES prepared from choline chloride and malonic acid (ChCl:MA) [57] (Scheme 11).

**Scheme 12:**
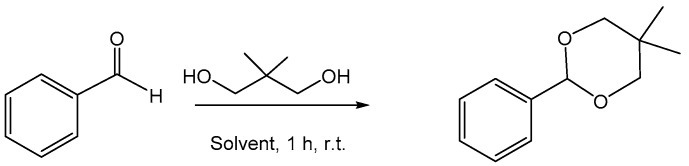
Acetalization reaction of benzaldehyde.

However, the described synthetic procedure shows the disadvantage of having to use high reaction temperatures. Therefore, developing even greener and safer technology is highly desirable. In this context, Arnodo et al. [58] investigated the effectiveness and feasibility of using acidic NADESs as solvents to perform acetalization reactions, trying to develop a synthetic process that can be used on a large scale and is recyclable. The effectiveness of the NADESs was investigated by evaluating the reaction conditions with benzaldehyde and neopentyl glycol as the model reagents. ChCl-based NADESs and glycerol and urea as the HBDs were tested, but the NADESs that showed greater effectiveness were the NADESs in which the HBD showed a particular acidity, such as malonic acid, oxalic acid and lactic acid. The reactions were tested at room temperature (Scheme 12).

The reactions were carried out using different molar quantities of diol and were compared to reactions carried out in classical solvents such as toluene, dichloromethane and acetonitrile. The results obtained showed the need to use NADESs, probably useful for capturing the water formed during the acetalization reaction, thus preventing the backshift of the equilibrium to the starting reagents. Furthermore, the importance of the acid component in the reaction process was demonstrated. The best results (87% yield) were obtained using ChCl/malonic acid at 1:1 mol/mol at room temperature with a molar ratio of aldehyde/diol of 1:1.2 mol/mol. The experiments were performed with recycled NADESs with various contents of residual water, and no significant variation in the yield of the product within this range of water content was observed.

## 3. Conclusions

In this manuscript, a collection of eco-sustainable protection reaction methods has been reported. In this regard, the use of deep eutectic solvents in different assisted systems for protection reactions with amine, hydroxyl and carbonyl groups has been described.

The methods reported in this review are all eco-sustainable synthetic processes that have made a major contribution, involving multifunctional molecules ideal for the synthesis of molecules of pharmacological interest. The use of green solvents, such as deep eutectic solvents, in the protection reactions of important functional groups presents significant advantages in the field of sustainability. The possibility of using eutectic mixtures made up of compounds of natural origin that are non-toxic and easily available, as well as the possibility of being able to recycle them and use them in subsequent steps, has revolutionized synthetic processes which, over the years, have been the main causes of pollution.

Many of the reactions reported in the literature demonstrate the efficiency of the recycled solvents when applied to the same synthetic processes. Also, not to be overlooked is the low cost of the components used to form the eutectic mixtures. It has also been demonstrated that, often, the eutectic solvent, in addition to its solvent function, is fundamental in the catalytic process reaction.

In fact, it has been seen how choline chloride has an important role in direct amidation reactions and is strongly important in the formation of DESs in situ, where one of the two components that forms the eutectic mixture is constituted of the starting product which will undergo the derivatization reaction.

These synthetic methods increase the level of sustainability of the entire synthetic process even further if we consider that the only waste product obtained at the end of the process is the useful component of the eutectic mixture not involved in the transformation process. The latter is easily recoverable for work-up at the end of the synthetic transformation and could be reusable.

It would be very interesting in the future to hypothesize and test new eutectic mixtures in which the component that must undergo protection is a natural product. This would involve the synthesis of potential drugs or new nutraceuticals so that their pharmacological and biological activities could be tested.
